# Case Report: Mimicking benignity: hepatic sinusoidal metastasis masquerading as diffuse liver disease in small cell lung cancer

**DOI:** 10.3389/fonc.2025.1655532

**Published:** 2025-08-19

**Authors:** Lujiao Chen, Lulin Chen, Jianfeng Yang, Minxia Yang

**Affiliations:** ^1^ Department of Radiology, Shaoxing People’s Hospital, Shaoxing, China; ^2^ Department of Ultrasound, Affiliated hospital of Shaoxing University, Shaoxing, China

**Keywords:** small cell lung cancer, diffuse intrahepatic metastasis, liver failure, treatment, case report

## Abstract

**Background:**

Small cell lung cancer is extremely aggressive. Although liver metastasis is common, cases of diffuse intra-sinusoidal metastasis leading to liver failure and death are quite rare.

**Case presentation:**

This paper reports a case of a 58-year-old male diagnosed with small cell lung cancer through a pathological biopsy, who died due to the rapid progression of liver failure caused by diffuse hepatic sinusoidal metastasis during subsequent treatment.

**Conclusions:**

Diffuse intra-sinusoidal liver metastasis originating from small cell lung cancer may serve as a clinically occult cause of liver failure. Such metastases progress rapidly, often leading to death within days to weeks. For patients with a history of malignant tumors, if imaging studies reveal diffuse liver lesions and rapidly progressing liver function abnormalities, there should be a high suspicion of diffuse hepatic sinusoidal metastasis. A timely liver biopsy should be performed to confirm the diagnosis, providing a theoretical basis for clinical diagnosis and treatment.

## Introduction

Small cell lung cancer (SCLC) accounts for approximately 13-15% of all lung cancer cases and is a highly lethal malignancy, with a 5-year survival rate of less than 7%. This subtype of lung cancer is characterized by high aggressiveness, rapid proliferation, and a propensity for distant metastasis (1, 2). SCLC is typically staged into two categories based on disease extent: limited-stage (LS)-SCLC and extensive-stage (ES)-SCLC (3). Approximately two-thirds of patients present with extensive-stage disease at initial diagnosis (4–6), with 17% exhibiting liver metastases (7, 8). Hepatic metastasis from small cell lung cancer typically presents as space-occupying lesions on imaging. Rarely, diffuse infiltration of the hepatic sinusoids by tumor cells can occur, rendering the lesions undetectable through imaging. Patients often present with hepatitis-like symptoms and rapidly progress to acute liver failure when metastatic cancer cells infiltrate the hepatic sinusoids rather than forming discrete masses. Sinusoidal metastasis within the liver is an uncommon condition observed in various malignancies, characterized by diffuse infiltration of the liver by metastatic foci.Currently, only a limited number of cases of diffuse intrahepatic metastasis (DIM) have been documented in the literature. Given its rarity and the absence of nodular lesions, the diagnosis of severe liver metastasis and the underlying cause of liver failure can be challenging at the clinical stage, often necessitating confirmation through histopathological examination. This report will detail a case of small cell lung cancer presenting with intrahepatic sinusoidal metastasis, offering insights for clinical diagnosis and treatment.

## Case report

**Figure 1:**
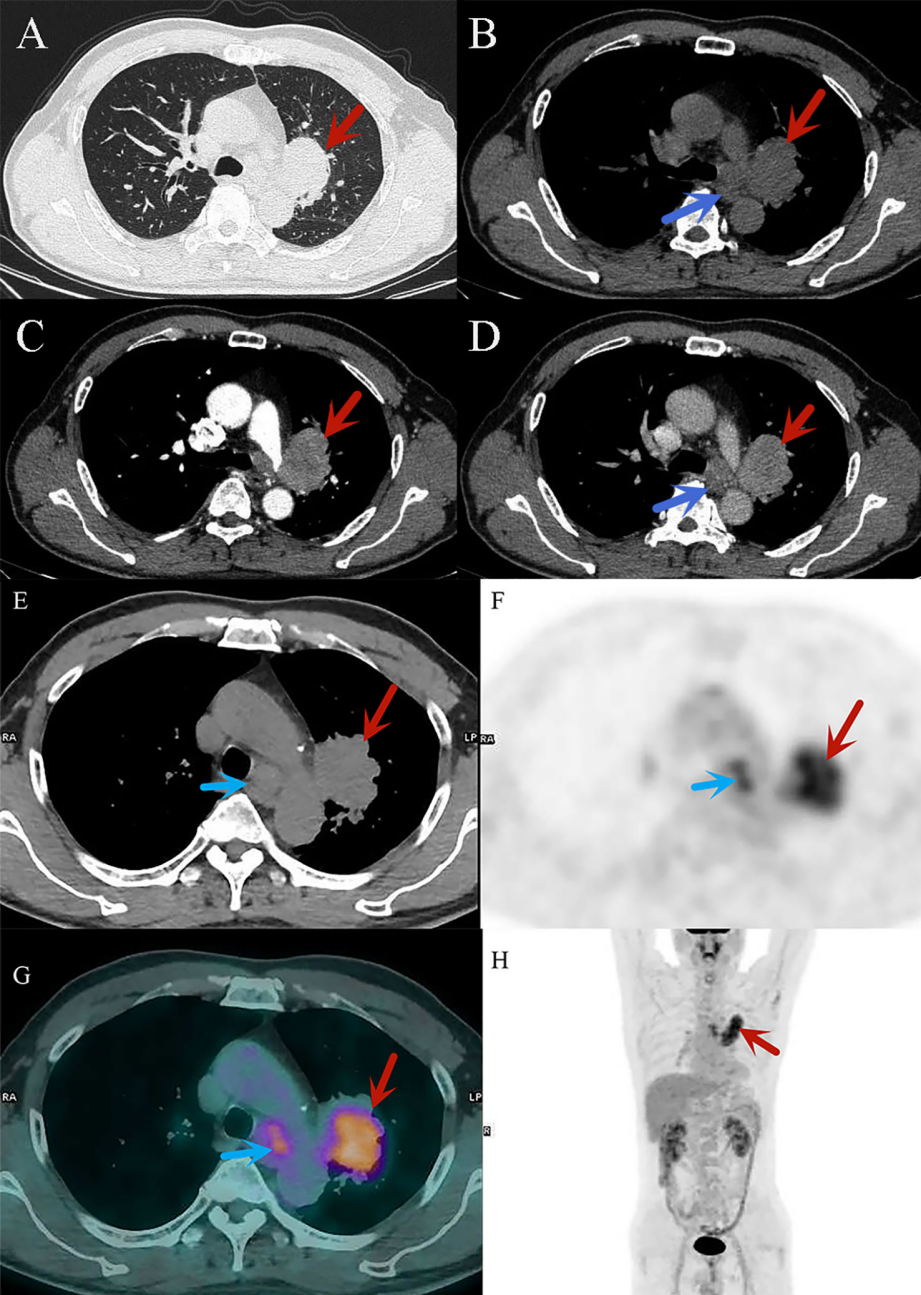
**(A–D)** CT diagnostic images of small cell lung cancer, **(A)** the lung window shows a mass in the upper lobe of the left lung (red arrow); **(B–D)** the mediastinal window reveals significant heterogeneous enhancement of the mass in the upper lobe of the left lung, with mediastinal lymph node enlargement (blue arrow). **(E–H)** 18F-FDG PET/CT images of small cell lung cancer. (**(E)**: CT image) The cancerous lesion in the upper lobe of the left lung (red arrow) and metastatic lymph nodes in the mediastinum (blue arrow). (**(F)**: PET image) The cancerous lesion in the upper lobe of the left lung (red arrow) and the mediastinal lymph nodes (blue arrow) exhibit high metabolic activity, appearing as areas of radioactive uptake. (**(G)**: PET/CT fusion image) Clearly shows the high metabolic lesions in the upper lobe of the left lung (red arrow) and the mediastinum (blue arrow), combining CT morphological information with PET metabolic data, with the active metabolic regions corresponding precisely to the morphological features. (**(H)**: PET/CT fusion image) High metabolic lesions can be observed in the thoracic region (red arrow), appearing as radioactive uptake foci distributed along the body’s longitudinal axis, indicating the location of the pathology.

**Figure 2:**
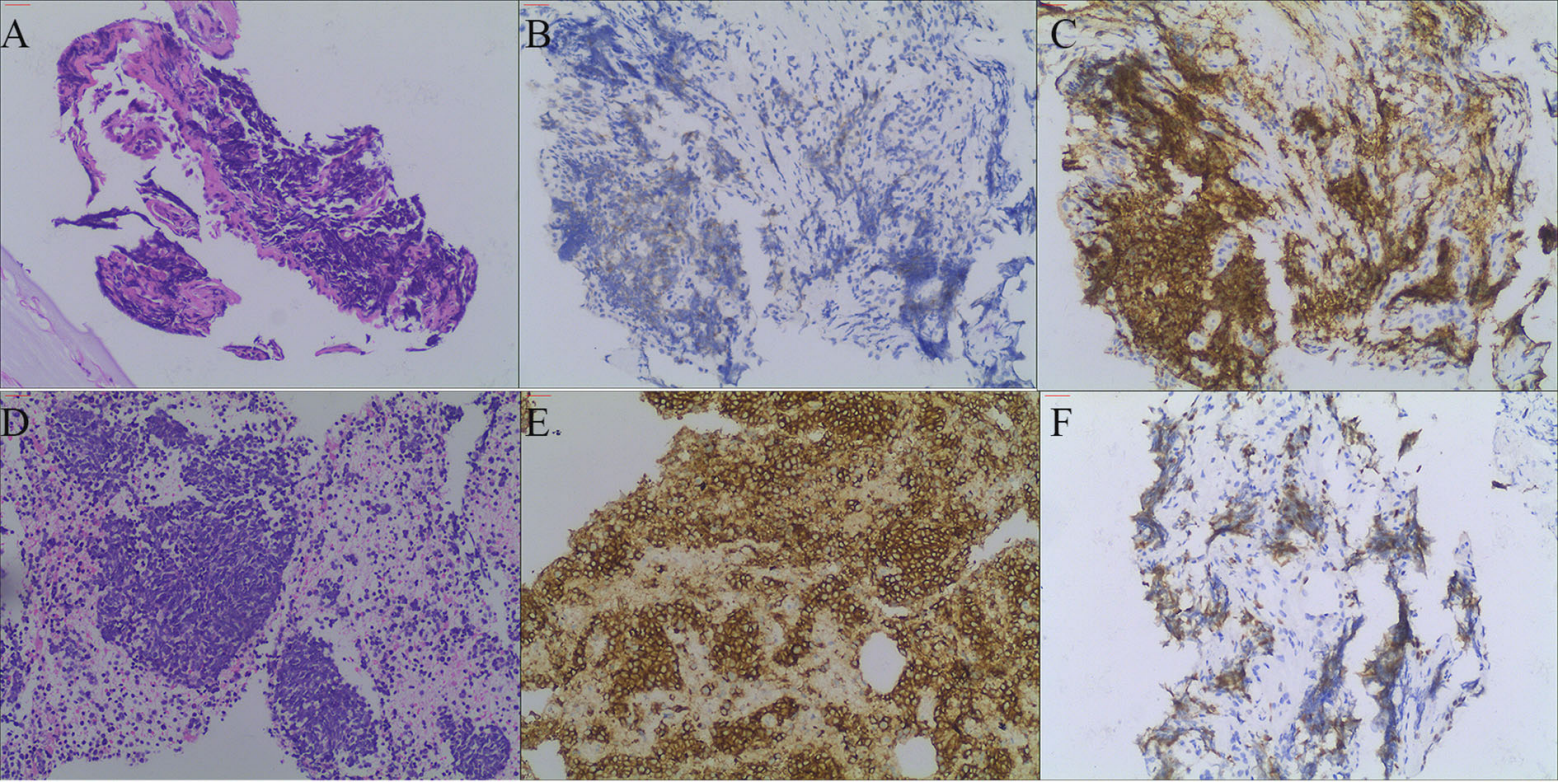
**(A)** shows HE staining of the pulmonary tumor lesion (magnification 100×), revealing small round cells with compressed deformation. **(B, C)** show CgA and CD56 immunohistochemical staining of the pulmonary tumor lesion (magnification 100×), both of which are positive. **(D)** shows HE staining of the lymph node (magnification 100×), revealing tumor cell infiltration. **(E, F)** show lymph node CD56 and Ki-67 immunohistochemical staining (magnification 100×), both of which are positive.

**Figure 3:**
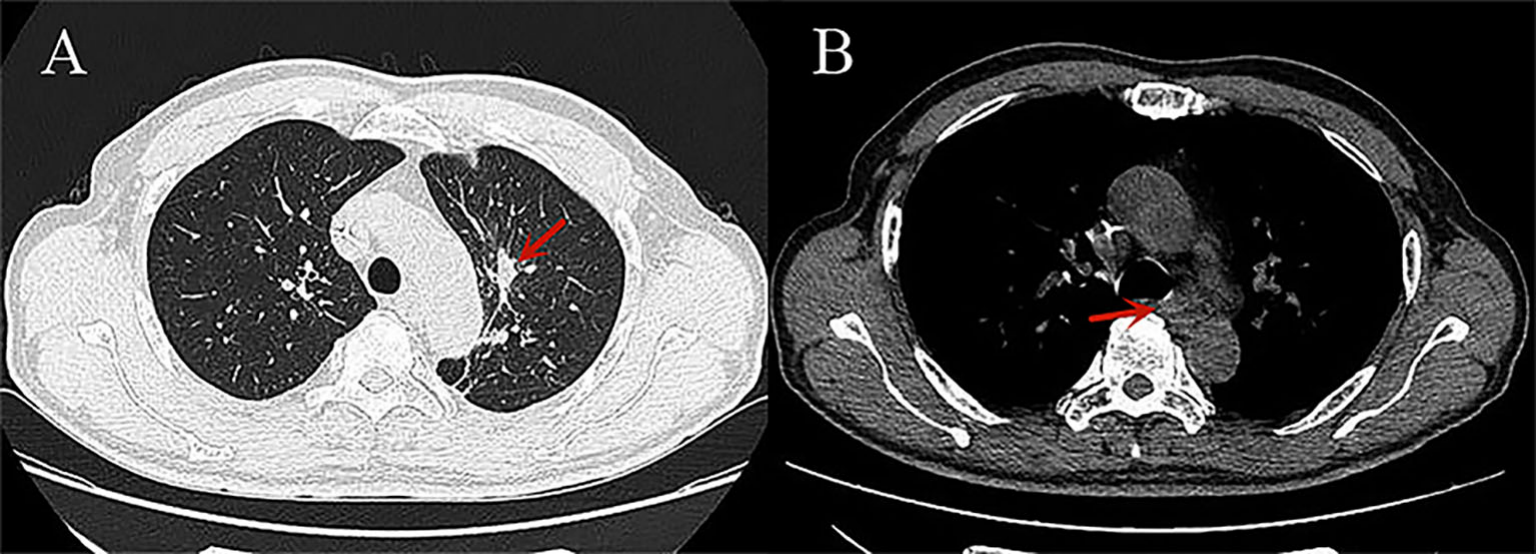
CT images of small cell lung cancer after treatment: **(A)** The lung window shows significant shrinkage of the cancerous lesion in the left upper lobe (red arrow); **(B)** The mediastinal window shows significant shrinkage of the metastatic lymph nodes in the mediastinum (red arrow).

A 58-year-old male presented to the Department of Cardiothoracic Surgery with complaints of chest tightness and cough. A chest CT scan with contrast revealed a space-occupying lesion in the left upper lobe, accompanied by mediastinal and left hilar lymphadenopathy (**Figures 1A–D**). Subsequent pathological biopsy confirmed small cell lung cancer (**Figures 2A–C**), with mediastinal lymph node biopsy indicating metastasis (**Figures 2D–F**). The patient underwent PET/CT, which did not reveal any distant metastasis (**Figures 1E–H**). According to the eighth edition of the International Association for the Study of Lung Cancer (IASLC) staging system, the TNM stage was determined to be IIIB (T4N2M0).Subsequently, the patient commenced first-line EP chemotherapy (Etoposide 0.18 g on days 1–5 plus Cisplatin 40 mg on days 1-3) on March 31, 2023, with each cycle lasting 21 days. During the third cycle of EP chemotherapy (May 13, 2023), concurrent definitive radiotherapy was administered (60 Gy in 30 fractions) on May 16, 2023. The radiation field encompassed the primary pulmonary tumor, ipsilateral hilar lymph nodes, and mediastinal lymph node stations. Following two cycles of concurrent chemoradiotherapy, a follow-up chest CT indicated a partial response (PR) (**Figure 3**, June 2, 2023). The patient continued with an additional two cycles of EP chemotherapy before cessation. Over the subsequent six months, no signs of recurrence were observed. However, follow-up examination results at six months revealed liver metastasis (**Supplementary Figure S1A**, 2023.12.19), followed by an IP regimen (irinotecan 110mg d1,8 + cisplatin 50mg d1,8) chemotherapy, administered every three weeks. After two cycles, upper abdominal MRI (**Supplementary Figure S1B**, 2024.02.05) demonstrated enlargement of the liver metastasis, prompting a switch to the IP regimen (irinotecan 110mg d1 + cisplatin 50mg d1) chemotherapy combined with Sluri(200mg d1) monoclonal antibody immunotherapy. After two cycles, upper abdominal MRI enhancement showed increased liver metastases (**Supplementary Figure S1C**, 2024.03.28). Consequently, the patient underwent hepatic artery embolization with chemotherapy drug perfusion (etoposide 100mg + carboplatin 100mg). One week later, anlotinib targeted therapy was initiated. After three weeks, the patient’s upper abdominal MRI showed continued increase in liver metastases (**Supplementary Figure S1D**, 2024.05.20).

**Figure 4:**
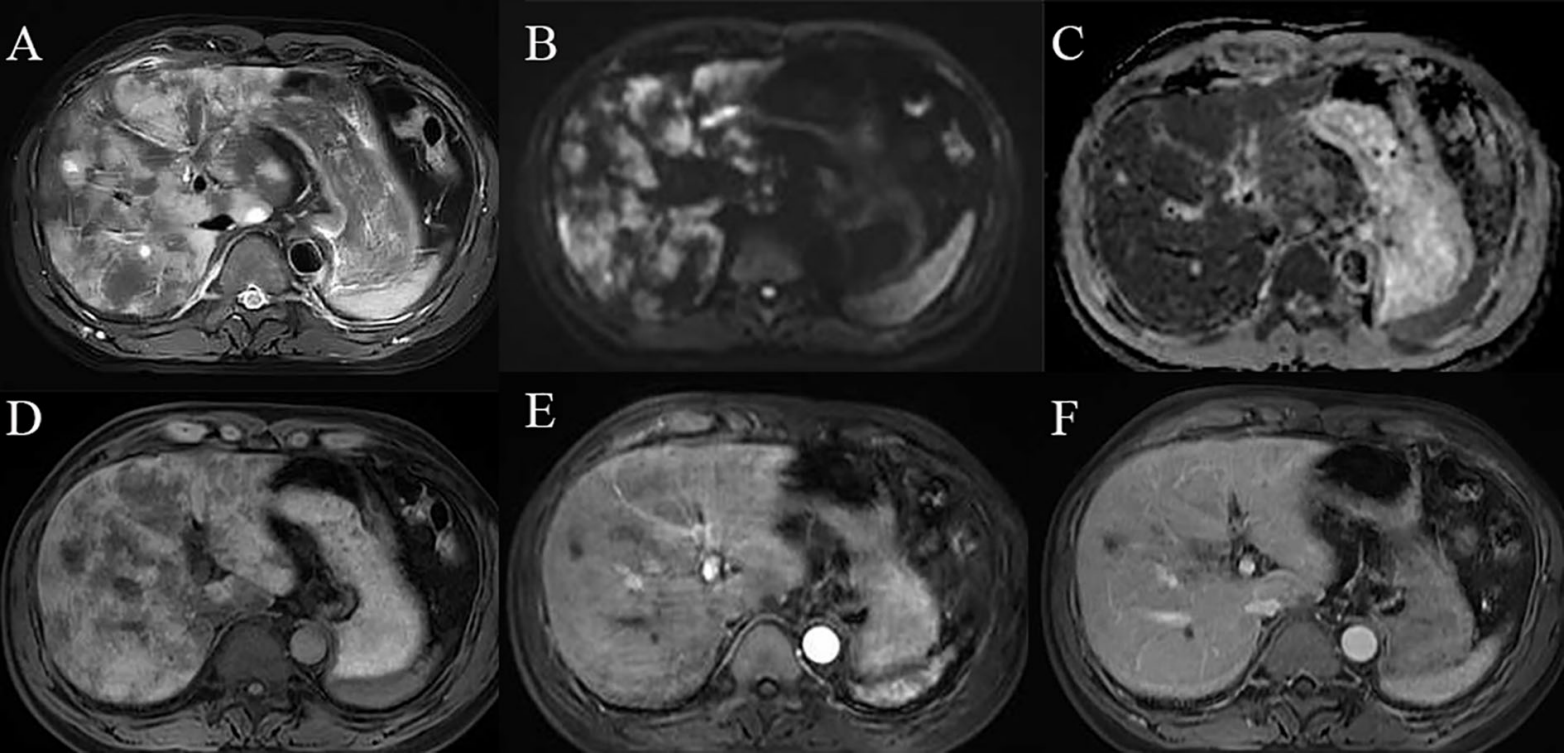
Diffuse hepatic sinusoidal metastases on MRI images: **(A)** T2-weighted imaging (T2WI) reveals diffuse hyperintensity within the liver;**(B, C)** Diffusion-weighted imaging (DWI) and apparent diffusion coefficient (ADC) demonstrate restricted diffusion of the lesions. **(D)** T1-weighted imaging (T1WI) shows diffuse hypointensity in the liver. **(E, F)** Contrast-enhanced scans do not exhibit significant enhancement of the lesions.

The patient’s intrahepatic metastases are persistently progressing, raising suspicion of a potential combined small cell lung carcinoma. Subsequently, the patient’s treatment regimen was altered to include targeted therapy, immunotherapy, and chemotherapy (Anlotinib 10mg d1-14,Q3W + Sulumzumab 200mg d1+ Albumin Paclitaxel 300mg d1,Q3W), administered every three weeks. Following two cycles, a follow-up upper abdominal MRI revealed a reduction in metastatic lesions (**Supplementary Figure S1E**, 2024.07.02), and treatment was continued. After an additional two cycles, a repeat upper abdominal MRI (**Figure 4**, 2024.08.12) demonstrated new diffuse lesions within the liver. Liver function tests indicated elevated levels of alanine aminotransferase (ALT), aspartate aminotransferase (AST), γ-glutamyl transferase (GGT), alkaline phosphatase, and total bilirubin (**Supplementary Table S1**). Given the possibility of drug-induced hepatitis, liver protection measures and intravenous methylprednisolone were initiated. However, the patient’s condition continued to deteriorate, prompting a liver biopsy. The biopsy results confirmed small cell carcinoma metastasis and cancer cell infiltration within the hepatic sinusoids (**Supplementary Figure S2**). Ultimately, the patient developed multi-organ failure and succumbed to the disease. (**Supplementary Figures S3**, **S4** provide a concise overview of the patient’s treatment timeline and the dynamic changes in specific biochemical indicators during the treatment period).

## Discussion

In the progression of small cell lung cancer, the incidence of secondary lesions within the liver parenchyma is notably high. Typically, patients present with multiple nodular lesions within the liver parenchyma. However, atypical presentations are observed in clinical practice. The tumor may infiltrate and diffusely spread along the hepatic sinusoids without forming a discrete mass, complicating accurate disease identification via imaging. This process can result in widespread tumor dissemination within the liver parenchyma, remaining radiologically occult until hepatic sinusoidal obstruction occurs, subsequently leading to acute liver failure. Indeed, this pattern of diffuse sinusoidal metastasis represents a frequent etiology of acute liver failure. While this metastatic pattern is more commonly associated with hematological malignancies, it is also linked to various solid tumors, including small cell lung cancer, breast cancer (9), gastric cancer (10), urethral cancer (11), and nasopharyngeal carcinoma (12).

This case involves a 58-year-old male patient diagnosed with small cell lung cancer. The patient’s clinical condition progressively deteriorated, with the gradual onset of liver failure symptoms. Abdominal magnetic resonance imaging revealed a typical mass of metastatic tumors in the early stages of the disease, progressing to diffuse liver lesions in later stages of treatment. This diffuse lesion is a common imaging manifestation of tumor sinus spread, generally attributed to the dissemination of tumor cells along the hepatic and portal veins, leading to intravascular stenosis, obstruction, and ischemia (13). It is noteworthy that, due to the invasion and obstruction of the hepatic sinusoids, the patient exhibited clinical features consistent with hepatic sinusoidal obstruction syndrome, which differed from the initial imaging findings of the metastatic lesions and led to a misdiagnosis of drug-induced hepatitis. Typically, liver metastasis presents as multiple tumor-like lesions; however, poorly differentiated tumors can diffusely spread within the hepatic sinusoids (14). This case mirrors the individual cases reported in the literature, demonstrating a mixed pattern of sinusoidal diffusion and hepatic parenchymal nodules. Previously, this mixed pattern has been observed in 1 case of pancreatic cancer (15), 2 cases of breast cancer (16, 17), and 3 cases of ocular melanoma (18).

This metastatic diffusion pattern is exceedingly rare, with limited reports available on related cases. In 1955, Watson (19) reported five instances of intrasinus metastasis, all originating from bronchogenic sources. Smith, in 1961, noted in a literature review that only 25 cases of intrasinus metastasis (DIM) had been previously documented (20). The hallmark of DIM is significant hepatomegaly, while generally preserving the liver’s architecture. Tumor cells diffusely infiltrate the hepatic sinusoids, rather than forming discrete mass lesions, and may invade hepatic and portal vein branches. In certain instances, the body’s pronounced connective tissue hyperplasia response to malignant cells can result in a cirrhotic-like liver appearance. Hepatocytes may ultimately succumb to compression atrophy and vascular infarction, precipitating rapid patient demise. Furthermore, studies suggest that tumor cell-released cytokines can disrupt bile ducts and induce portal fibrosis, subsequently leading to sinusoidal microcirculation obstruction and hepatocyte ischemia (21, 22). The absence of pathognomonic symptoms and imaging characteristics often complicates diagnosis prior to pathological biopsy.

In radiological imaging, diffuse intrahepatic sinusoidal metastases on computed tomography (CT) typically manifest as diffuse hypodensity with heterogeneous attenuation, often lacking distinct nodular or mass-like lesions, although hepatomegaly may be present—potentially due to diffuse infiltration of tumor cells within the hepatic sinusoids. During the arterial phase of contrast-enhanced CT, tumor cell infiltration causes diffuse sinusoidal filling, resulting in heterogeneous mild enhancement of the hepatic parenchyma without clear nodular enhancement. In the portal venous phase, tumor cell obstruction within the sinusoids impairs blood flow, leading to decreased enhancement relative to normal liver tissue, presenting as hypoperfusion or patchy perfusion defects. Magnetic resonance imaging (MRI) commonly demonstrates a diffuse reduction in signal intensity on T1-weighted images, with a corresponding diffuse increase on T2-weighted and fat-suppressed sequences, producing a “snowflake” or “frosted glass” appearance—likely attributable to sinusoidal tumor infiltration and edema. Diffusion-weighted imaging (DWI) often shows restricted diffusion. During arterial phase contrast-enhanced MRI, mild heterogeneous enhancement may occur, possibly due to uneven contrast distribution caused by sinusoidal tumor infiltration. In the portal venous and delayed phases, enhancement remains inferior to that of normal liver tissue, presenting as “map-like” or “reticulated” hypointense areas, which should be differentiated from hepatic injury induced by chemotherapeutic agents. Chemotherapy-related hepatic injury typically manifests as steatosis and vascular damage (hepatic sinusoidal syndrome, SOS), with CT findings including diffuse hepatomegaly, heterogeneous hypodensity of the parenchyma, peripheral distribution of enhancement—predominantly affecting the right lobe—forming a “claw-like” pattern, along with inferior vena cava obstruction and poorly defined hepatic veins. The degree of enhancement heterogeneity correlates with chemotherapy cycles and the extent of sinusoidal injury; more pronounced heterogeneity indicates more severe sinusoidal damage histologically. Severe cases may present with moderate ascites, gallbladder wall edema, splenomegaly, and gastrointestinal wall edema. MRI with contrast may reveal characteristic features such as patchy or diffuse reticulated hypointensity in the liver parenchyma and thickening of Glisson’s capsule. The cases described herein resemble hepatic injury secondary to chemotherapy, with atypical diffuse lesions observed on abdominal CT and MRI, posing diagnostic challenges. Confirmatory diagnosis was established via liver biopsy, which demonstrated positivity for synaptophysin (Syn), chromogranin A (CgA), CD56, and Ki-67, consistent with metastatic small cell lung carcinoma.

At the initial diagnosis of metastasis in this study’s patients, secondary lesions within the liver parenchyma presented with nodular and mass-like features, and the metastasis remained refractory to various treatments. The histological transformation of lung cancer is a complex process, and its underlying mechanisms are not fully elucidated. One cancer cell type may transform into another with distinct histological characteristics. Studies (23) have confirmed that non-small cell lung cancer (NSCLC) can transform into small cell lung cancer (SCLC), and both cancer cell types may coexist within the same patient.Chen et al. (24) reported a case of an NSCLC patient who developed drug resistance due to gene mutation during treatment and subsequently transformed into SCLC. Compound small cell lung cancer (C-SCLC) is defined by the WHO as the combination of small cell lung cancer with any histological type of non-small cell lung cancer, including adenocarcinoma (ADC), squamous cell carcinoma (SCC), large cell carcinoma (LCC), large cell neuroendocrine carcinoma (LCNEC), and rare spindle cell carcinoma or giant cell carcinoma. A 2018 study indicated that (25) C-SCLC cases account for approximately 5%-10% of all SCLC cases. However, there is currently insufficient literature evidence to support the transformation of small cell lung cancer into non-small cell lung cancer. Based on these findings, we hypothesize the patient may have a compound small cell lung cancer and propose the addition of albumin-bound paclitaxel to the treatment regimen. Initially, a significant therapeutic response was observed, with a reduction in the size of the patient’s metastatic tumors. Unfortunately, after four cycles of combination therapy with albumin-bound paclitaxel, liver imaging revealed diffuse, amorphous lesions accompanied by abnormal liver function.

Compound small cell lung cancer (C-SCLC) represents a distinct subtype of small cell lung cancer (SCLC). Tumor lesions predominantly affect the central airway, frequently accompanied by mediastinal lymph node involvement. Currently, the cellular origin, biological characteristics, and other aspects remain incompletely elucidated, lacking robust, high-level evidence-based medical support. Furthermore, C-SCLC is characterized by a propensity for early metastasis and a poor prognosis, particularly in patients with extensive-stage disease. These clinical features are highly consistent with this case. Unfortunately, due to various reasons, the patient was unable to undergo genetic testing for a definitive diagnosis. The disease pattern of diffuse intrahepatic metastasis often presents with occult imaging findings in cases of acute liver failure, frequently associated with rapid progression and a poor prognosis. Due to the invasive nature of tumor diffusion, the disease often manifests with non-specific imaging signs. Therefore, this metastatic pattern warrants heightened vigilance in clinical practice, and diagnosis should be confirmed promptly via liver biopsy. In this instance, imaging examinations not only clearly demonstrated invasive hepatic lesions but also effectively assessed disease severity, providing a crucial baseline reference for treatment planning. Early identification of such metastatic patterns and timely intervention are key to improving patient prognosis and prolonging survival.

## Conclusion

In summary, diffuse intrahepatic metastasis from small cell lung cancer may represent an occult etiology of liver failure. This form of metastasis exhibits rapid progression, often leading to patient demise within a timeframe of days to weeks. Due to the homogeneous infiltration pattern within the liver, imaging studies may be prone to misdiagnosis. In this instance, we observed the potential for a composite small cell lung cancer, underscoring the importance of incorporating genomic sequencing during treatment, particularly in patients exhibiting disease progression, to facilitate the early identification of potential therapeutic targets. Repeat biopsies may be considered, and treatment strategies should be dynamically adjusted based on molecular pathological findings to enable individualized precision medicine. Furthermore, in patients with a history of malignancy, the presence of diffuse hepatic lesions on imaging, coupled with the rapid progression of liver dysfunction, necessitates a high index of suspicion for diffuse intrahepatic metastasis, warranting timely confirmation via liver biopsy. Future investigations are warranted to elucidate the underlying biological mechanisms of this specific metastatic pattern, thereby informing clinical diagnosis and management.

## Data Availability

The original contributions presented in the study are included in the article/**Supplementary Material**. Further inquiries can be directed to the corresponding author.
