# Supplementary material for: Case Report: Mimicking benignity: hepatic sinusoidal metastasis masquerading as diffuse liver disease in small cell lung cancer
Source: Front Oncol. 2025 Aug 19;15:1655532. doi: 10.3389/fonc.2025.1655532 (PMC12401973; doi:10.3389/fonc.2025.1655532)
Supplement: Supplementary file 1 [file DataSheet1.docx]

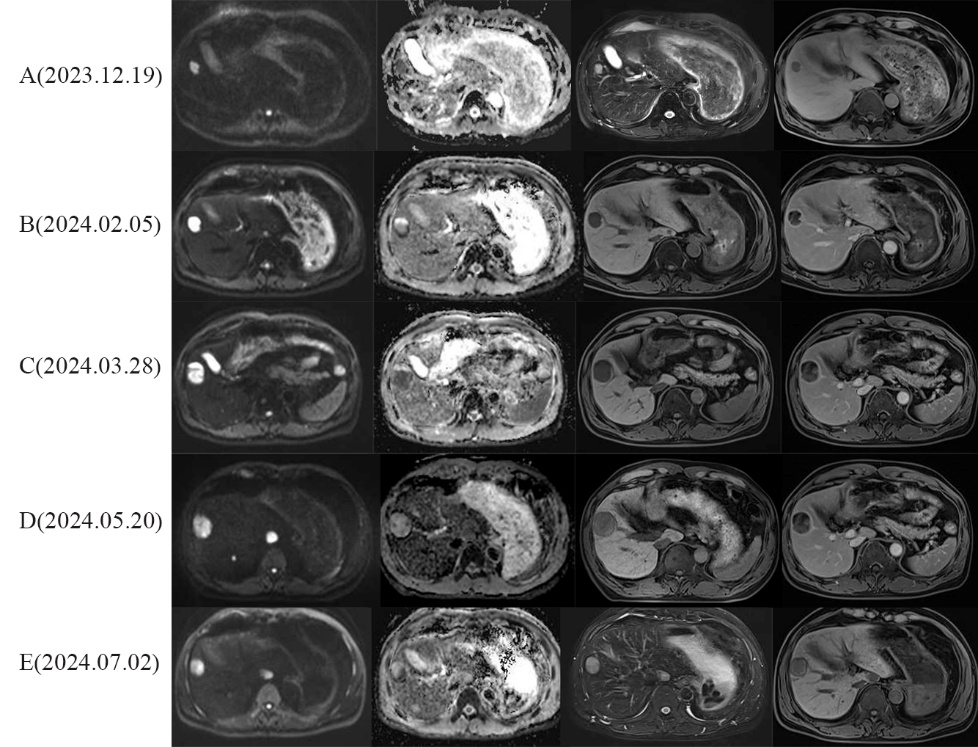


**Fig. S1** Changes in liver metastases on abdominal MRI. (A) Initial image of liver metastases; (B) Image of liver metastases after treatment with the IP regimen; (C) Image of liver metastases with disease progression after chemotherapy with the IP regimen plus immunotherapy with slurilumab; (D) Image of liver metastases after hepatic artery embolization plus chemotherapy drug infusion (etoposide plus carboplatin) and targeted therapy with anlotinib; (E) Image of liver metastases showing reduction in size after treatment with anlotinib + slurilumab + albumin-bound paclitaxel.


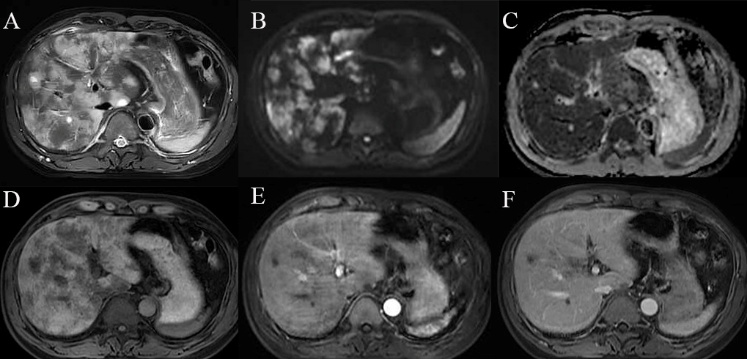


**Fig.S2** MR images of diffuse metastatic tumors in the hepatic sinusoids (A) T2-weighted images (T2WI) show diffuse high signal intensity within the liver. DWI (B) and ADC (C) show restricted diffusion in the lesions. (D) T1-weighted images (T1WI) show diffuse low signal intensity within the liver. (E/F) Enhanced scans show no significant enhancement in the lesions.


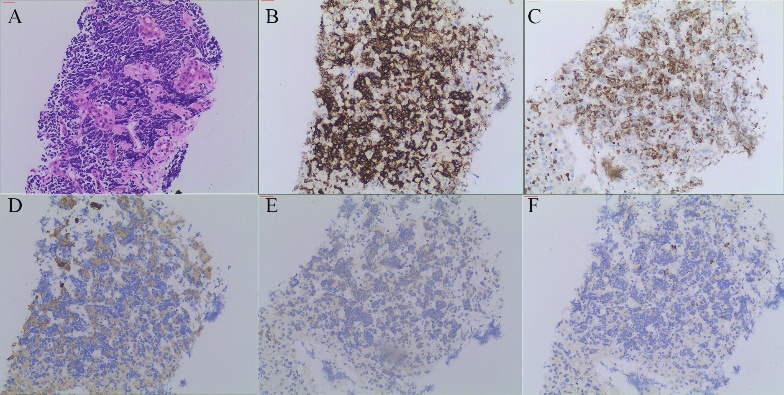


**Fig.S3** Liver biopsy via liver puncture, magnification ×100. Figure A shows HE staining, revealing tumor cells infiltrating hepatic sinusoids or distributed in patches. Figures B and C show CD56 and INSM1 immunohistochemical staining, both of which are positive. Figures D to F show CKpan, Syn, and CgA immunohistochemical staining, all of which are partially weakly positive.

**Table S1：Abnormal changes in the patient's liver function indicators**

| Parameter | Normal value or range | 2024.8.12 | 2024.8.18 | 2024.08.21 | 2024.8.23 | 2024.8.26 | 2024.8.29 |
| --- | --- | --- | --- | --- | --- | --- | --- |
| ALT (U/L) | 9.0-50.0 | 91.3 | 208.0 | 123 | 91 | 98 | 128 |
| AST (U/L) | 15.0-40.0 | 88.3 | 144.0 | 100 | 92 | 142 | 214 |
| GGT (U/L) | 10.0-60.0 | 132.1 | 305.0 | 321 | 409 | 501 | 706 |
| Alkaline phosphatase (U/L) | 45.0-125.0 | 103.0 | 101.0 | - | 161 | 177 | 232 |
| Total bilirubin (µmol/L) | 3.4-20.5 | 13.0 | 11.6 | 29.3 | 65.2 | 85.8 | 199 |


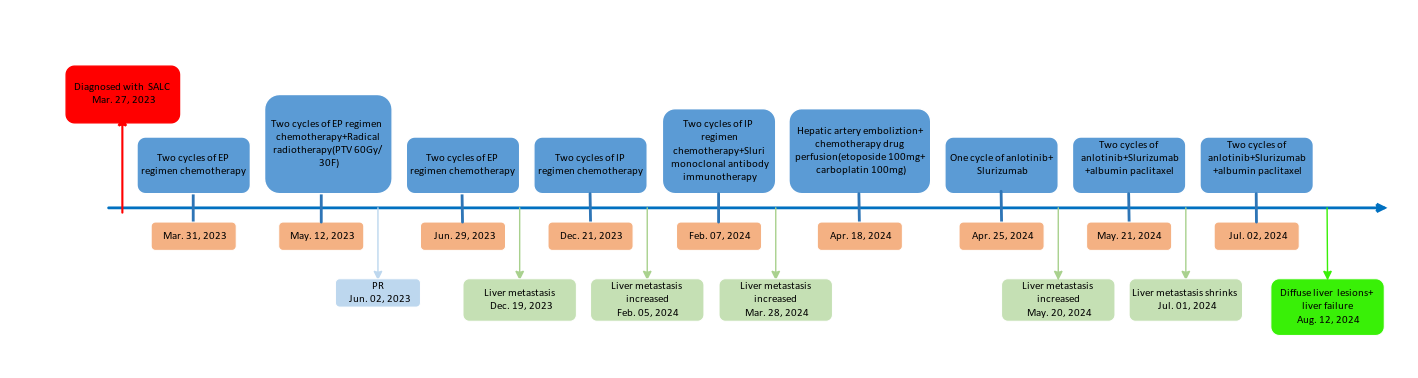


**Fig.S4** Patient treatment timeline
